# High-Fat Diet Augments the Effect of Alcohol on Skeletal Muscle Mitochondrial Dysfunction in Mice

**DOI:** 10.3390/nu14051016

**Published:** 2022-02-28

**Authors:** Ahmed Ismaeel, Joseph A. Laudato, Emma Fletcher, Evlampia Papoutsi, Abigail Tice, Lara S. Hwa, Dimitrios Miserlis, Athanasios Z. Jamurtas, Jennifer Steiner, Panagiotis Koutakis

**Affiliations:** 1 Department of Biology, Baylor University, Waco, TX 76798, USA; ahmed_ismaeel@baylor.edu (A.I.); emma_fletcher1@baylor.edu (E.F.); evlampia_papoutsi@baylor.edu (E.P.); 2 Department of Nutrition and Integrative Physiology, Florida State University, Tallahassee, FL 32304, USA; jal19@my.fsu.edu (J.A.L.); at19r@my.fsu.edu (A.T.); jsteiner2@fsu.edu (J.S.); 3 Department of Psychology and Neuroscience, Baylor University, Waco, TX 76798, USA; lara_hwa@baylor.edu; 4 Department of Surgery, University of Texas Health Science Center San Antonio, San Antonio, TX 78229, USA; miserlisd@uthsca.edu; 5 Department of Physical Education and Sport Sciences, University of Thessaly, 42100 Trikala, Greece; ajamurt@pe.uth.gr; 6 Department of Nutrition and Dietetics, University of Thessaly, 42100 Trikala, Greece

**Keywords:** alcohol, mitochondrial function, oxidative stress, aldehyde dehydrogenase 2

## Abstract

Previous studies have shown that chronic heavy alcohol consumption and consumption of a high-fat (HF) diet can independently contribute to skeletal muscle oxidative stress and mitochondrial dysfunction, yet the concurrent effect of these risk factors remains unclear. We aimed to assess the effect of alcohol and different dietary compositions on mitochondrial activity and oxidative stress markers. Male and female mice were randomized to an alcohol (EtOH)-free HF diet, a HF + EtOH diet, or a low-Fat (LF) + EtOH diet for 6 weeks. At the end of the study, electron transport chain complex activity and expression as well as antioxidant activity and expression, were measured in skeletal muscles. Complex I and III activity were diminished in muscles of mice fed a HF + EtOH diet relative to the EtOH-free HF diet. Lipid peroxidation was elevated, and antioxidant activity was diminished, in muscles of mice fed a HF + EtOH diet as well. Consumption of a HF diet may exacerbate the negative effects of alcohol on skeletal muscle mitochondrial health and oxidative stress.

## 1. Introduction

The National Institute on Alcohol Abuse and Alcoholism (NIAAA) defines heavy alcohol use as consumption of >3 or 4 drinks per day, or >7 or 14 drinks per week, for females and males, respectively [1]. Based on this definition, data from the National Health Interview Survey found that 5.1% of the U.S. population aged 18 and older engaged in heavy alcohol use in 2018 [1]. Ample amounts of clinical and experimental evidence suggest associations between heavy alcohol use (also defined as heavy drinking) and a wide range of medical conditions [2]. In fact, heavy alcohol use has been linked to at least 60 acute and chronic diseases [2].

Chronic heavy alcohol consumption affects health via various mechanisms, including adverse effects on several organs and tissues. These effects have mostly been studied in the brain, heart, liver, and pancreas [3], although skeletal muscle dysfunction, referred to as alcoholic myopathy, can occur in up to 50% of alcoholic patients [4]. The most well-studied mechanism believed to contribute to this skeletal muscle dysfunction is alcohol-induced impairment in muscle protein synthesis, which is thought to occur mainly via decreased activation of the mammalian target of rapamycin (mTOR) signaling pathway [5]. Beyond imbalanced protein metabolism, oxidative stress and mitochondrial dysfunction may also play roles as pathophysiologic mechanisms underlying alcohol-mediated loss of muscle mass and strength [6,7]. Notably, chronic alcohol feeding in mice and rats perturbs antioxidant activity, increases markers of oxidative stress, and reduces mitochondrial respiratory rates [8,9,10,11]. RNA sequencing and proteomic studies have identified that ethanol treatment in myotubes induces an upregulation in superoxide degradation pathways and a decrease in mitochondrial oxidative phosphorylation components [7]. Furthermore, high-resolution respirometry experiments show impaired respiration and increased reactive oxygen species (ROS) production in alcohol-treated myotubes and in skeletal muscle fibers from alcohol-fed mice [7].

Another modifiable health risk factor is poor diet [12]. In fact, consumption of a high-fat (HF) diet, consisting of ≥35% of total calories derived from fats, is associated with a myriad of diseases and conditions, including cardiovascular disease, obesity, diabetes, and cancer [13]. Like alcohol consumption, HF diet consumption has been shown to manifest in impaired mitochondrial respiration and increased ROS production [14,15].

As a result of the large energy requirement of skeletal muscle, skeletal muscle health is dependent on the optimal function of mitochondria [16,17]. Mitochondrial ATP production is driven by electron transfer through the electron transport chain, and decreased activity or dysfunction of the electron transport chain complexes has emerged as a central factor in several muscular diseases [18]. In addition to being the main suppliers of cellular energy, mitochondria also generate the majority of cellular ROS, with Complexes I and III having the greatest contribution [19,20]. In normal conditions, antioxidant defenses balance the increase in ROS by neutralizing free radicals; however, under pathological conditions, an imbalance leads to oxidative stress [21]. Importantly, oxidative stress is associated with various myopathies as excess ROS can act as a catabolic signal leading to muscle atrophy [22].

Due to shared and distinct mechanisms in alcohol and HF diet-induced mitochondrial dysfunction, there has been growing interest in studying their interactive and combined actions [23,24,25]. Interestingly, HF diet intake has been shown to exacerbate alcohol-induced effects on cardiac function [26], wound healing [27], brain function and behavior [28,29], and liver disease [30,31]. However, the concurrent effect of the two modifiable risk factors on skeletal muscle mitochondrial dysfunction remains unclear. Thus, the primary purpose of the present investigation was to observe how differences in macronutrient composition influence alcohol’s chronic effects on skeletal muscle mitochondrial electron transport chain activity in mice. These studies also assessed the effects of alcohol and different dietary compositions on antioxidant and oxidative stress measures.

## 2. Materials and Methods

### 2.1. Animals

Male (*n* = 13) and Female (*n* = 10) C57BL/6Hsd mice 10–12 weeks of age were purchased from Envigo (Indianapolis, IN, USA). Upon arrival, all mice were individually housed in the Biomedical Research Facility (BRF) vivarium at Florida State University (FSU) for at least 1 week prior to the start of the experiment. Mice were housed in a temperature-controlled (25 °C) environment on a 12:12 light/dark cycle (5 a.m.:5 p.m.). All procedures were approved by Animal Care and Use Committee at Florida State University and conformed to AVMA guidelines.

### 2.2. Alcohol and Dietary Treatments 

Mice within each sex were randomized to one of three dietary treatment groups to determine the effects of different macronutrient compositions on mitochondrial dysfunction induced by chronic alcohol (EtOH) intake. Experimental groups included EtOH-free HF, HF + EtOH, or low-Fat (LF) + EtOH. All diets were purchased from Dyets Inc (Bethlehem, PA, USA). EtOH-free HF mice were pair-fed a modified Lieber-DeCarli liquid diet (#710260) by adding maltose dextrin instead of ethanol to replace the alcohol-derived calories of the other treatment groups so that all diets were isocaloric by volume. The Lieber-DeCarli liquid diet is a well-established technique for alcohol feeding to rodents [32,33]. HF + EtOH mice consumed, ad libitum, the regular Lieber-DeCarli liquid diet (#710260); thus, the EtOH-free HF diet and HF + EtOH diet were matched with respect to fat content and only differed by alcohol (and consequently, carbohydrate) content. LF + EtOH mice consumed the low-fat Lieber-DeCarli liquid diet (#710261). The concentration of EtOH within the liquid diets was increased in a stepwise manner. The mice were allowed 5 days to acclimate to the control liquid diet containing 0% alcohol, after which the concentration of alcohol was increased to 6% of total kcal for 2 days, and then 12% of total kcals for another 2 days. Following this, the alcohol concentration was increased to 22% of kcals, and 27% of kcals, for 7 days each, prior to maintenance at 32% of kcals for the remaining 24 days of the experiment (6-week intervention total).

All diets were isocaloric and had identical vitamin and mineral compositions. The macronutrient breakdown of the diets is presented in Table 1. The ingredient breakdown of the diets is provided in Supplemental Table S1.

### 2.3. Tissue Collection

Mice were anesthetized via isoflurane inhalation, and the gastrocnemius muscle was removed. While still deeply anesthetized, the heart was removed for euthanasia. Gastrocnemius muscles were flash frozen in liquid nitrogen and then stored at −80 °C until further analysis. Tibias were isolated at sacrifice, and their length was measured with calipers.

### 2.4. Myoglobin Concentration

Commercial myoglobin enzyme-linked immunosorbent assay (ELISA) microwell strip plate kits were used for the determination of myoglobin in plasma and gastrocnemius tissue homogenates (Abnova, KA2473, Taipei, Taiwan). Leakage of myoglobin from skeletal muscle into circulation provides supportive evidence for muscle damage and is a marker of metabolic myopathies [34,35]. The technique used for myoglobin detection is based on a sandwich ELISA, using a monoclonal anti-myoglobin antibody for solid phase immobilization and a polyclonal horseradish peroxidase (HRP)-conjugated anti-myoglobin antibody for detection. The kits showed an intra-assay precision of 6.1% coefficient of variation (CV) and an inter-assay precision CV of 4.8%. All samples were thawed to room temperature prior to use. Plasma samples were diluted two-fold prior to testing, and muscle samples were diluted 20-fold. All samples, controls, and standards were assayed in duplicate. The optical density of the wells was determined using a Varioskan LUX Multimode Microplate Reader (Thermo Fisher, Waltham, MA, USA) set to 450 nm.

### 2.5. Electron Transport Chain Complex Activity

The activity of Complex I was determined in isolated mitochondria from mouse gastrocnemius muscles using a commercial assay kit (Sigma-Aldrich, MAK359, St. Louis, MO, USA). Mitochondria were isolated by differential centrifugation using mitochondrial buffer consisting of 0.6 M sucrose, 0.8% bovine serum albumin (BSA), and 160 mM 4-(2-hydroxyethyl)-1-piperazineethanesulfonic acid (HEPES), pH 7.4 (700× *g* for pelleting nonmitochondrial myofibrillar proteins, nuclei, and cellular components, followed by centrifugation of supernatant at 10,500× *g* to pellet mitochondria). Complex I activity catalytically converts decylubiquinone to decylubiquinol. Thus, by using a dye that accepts electrons from decylubiquinol, total catalytic activity is determined colorimetrically and compared to a standard curve of Complex I dye standards. Catalytic activity of samples was determined in the presence and absence of the Complex I inhibitor rotenone. The optical density of the wells was determined using a Varioskan LUX Multimode Microplate Reader set to 600 nm for 5 minutes. Catalytic activity was then determined as the change in reduced dye concentration over 5 minutes, normalized to protein content. Net Complex I activity was then determined by subtraction of the catalytic activity in the reaction with rotenone from the activity in the reaction without rotenone. All samples were run in duplicate, and results were averaged.

Complex III activity was similarly assayed using a commercial kit (Sigma-Aldrich, MAK360, St. Louis, MO, USA). Complex III transfers electrons from ubiquinol to cytochrome c; therefore, this assay uses the absorbance of reduced cytochrome c (550 nm) to determine Complex III activity. The absorbance is compared to a standard curve of prepared cytochrome c standards. The optical density of the wells was determined using a Varioskan LUX Multimode Microplate Reader for 5 minutes. Catalytic activity was calculated as the change in reduced cytochrome c during the 5 minutes, normalized to protein content. Net Complex III activity was then determined by subtraction of the activity in sample reactions with antimycin A (Complex III inhibitor) from reactions without antimycin A. All samples were run in duplicate, and results were averaged.

### 2.6. Oxidative Stress and Antioxidant Markers

A Lipid Peroxidation Assay Kit (Abcam, ab118970, Cambridge, UK) was used for the colorimetric detection of the lipid peroxidation marker malondialdehyde (MDA) in gastrocnemius muscle homogenates by reaction with thiobarbituric acid (TBA). All samples, controls, and standards were assayed in duplicate. The intra-assay variation was 4% CV. The optical density of the wells was determined using a Varioskan LUX Multimode Microplate Reader set to 532 nm. All samples were run in duplicate, and results were averaged.

A Superoxide Dismutase (SOD) Activity Assay Kit (Sigma-Aldrich, CS0009, St. Louis, MO, USA) was used to measure SOD activity in muscle homogenates (20 µg protein per sample). This kit uses xanthine oxidase to produce superoxide anions, which reacts with a dye to yield color at 450 nm. Thus, SOD activity was determined by monitoring the decrease in color signal, which is proportional to decreased superoxide anions, relative to an SOD enzyme standard. All samples and standards were run in duplicate, and the optical density of wells was determined using a Varioskan LUX Multimode Microplate Reader set to 450 nm. SOD activity is expressed as units of activity per mL (U/mL).

A Glutathione Peroxidase (GPx) Assay Kit (Abcam, ab102530, Cambridge, UK) was used for the quantification of the activity of glutathione-dependent peroxidases as a marker of antioxidant activity. Specifically, NADPH consumption during the reduction of oxidized glutathione by glutathione reductase was detected colorimetrically. All samples, controls, and standards were assayed in duplicate. The intra-assay variation was 6% CV. The optical density of the wells was determined using a Varioskan LUX Multimode Microplate Reader set to 340 nm. All samples were run in duplicate, and results were averaged.

### 2.7. Gene Expression

Total RNA was isolated from gastrocnemius samples using a commercial RNA spin-column format purification kit (Direct-zol RNA Microprep, Zymo Research, R2061, Irvine, CA, USA), followed by cDNA synthesis using an iScript Advanced cDNA Synthesis Kit (Bio-Rad Laboratories, 1725037, Hercules, CA, USA). Real-time PCR was used to determine the relative expression of the following antioxidant genes: Glutamate-Cysteine Ligase Catalytic Subunit (GCLC), Glutamate-Cysteine Ligase Modifier Subunit (GCLM), Heme Oxygenase 1 (HMOX1), Kelch-like ECH-associated protein 1 (Keap1), Nuclear factor-erythroid factor 2-related factor 2 (Nrf2), Peroxiredoxin 6 (PRDX6), and Cytochrome P450 Oxidoreductase (POR), and the housekeeping gene Beta-2 microglobulin (B2M) (Integrated DNA Technologies, San Diego, CA, USA). Table 2 lists the primer pair sequences. PCR reactions used a mixture of forward and reverse primers at a final concentration of 250 nM and 1x SsoAdvanced Universal SYBR Green Supermix (Bio-Rad Laboratories, Hercules, CA, USA), run on a CFX Opus Real-Time PCR System (Bio-Rad Laboratories, Hercules, CA, USA). The quantification cycle (Cq) of target genes was normalized to the reference gene (B2M), and relative normalized expression was calculated using the 2^(-delta delta Cq) method. All samples were run in triplicate, and results were averaged.

### 2.8. Protein Expression

Muscles were homogenized in ice-cold lysis buffer (50 mM Tris-HCl at pH 7.4, 150 mM NaCl, 1% NP-40, 0.25% Na-deoxycholate, 0.1% SDS, and 1x protease inhibitor cocktail) for protein isolation. Protein samples were mixed with 4x Laemmli Sample Buffer and 2-mercaptoethanol reducing agent (Bio-Rad Laboratories, Hercules, CA, USA), and then using NuPAGE 4–20% Criterion TGX gels, equal amounts of protein (10 µg) were separated using electrophoresis in a Criterion Cell Tank (Bio-Rad Laboratories, Hercules, CA, USA) for western blotting. Proteins were transferred to Immobilon-P polyvinylidene difluoride (PVDF) transfer membranes (MilliporeSigma, Burlington, MA, USA) and incubated in primary antibodies (OXPHOS rodent: Invitrogen 45–8099, 1:250 dilution; ALDH2: Invitrogen PA5-78757, 1:1000 dilution; AO: Abcam ab92519, 1:500 dilution; Nrf2: Cell Signaling 127215, 1:1000 dilution; and HMOX-1: Invitrogen MA5-31557, 1:500 dilution), followed by incubation with appropriate HRP-conjugated secondary antibody (Goat anti-Mouse IgG (H+L): Invitrogen g-21040 and Goat anti-Rabbit IgG (H+L): Invitrogen 31462, Invitrogen, Waltham, MA, USA). Membranes were visualized using Clarity ECL Substrate (Bio-Rad Laboratories, Hercules, CA, USA), and bands were detected using the ChemiDoc MP Imaging System (Bio-Rad Laboratories, Hercules, CA, USA). Band intensities were quantified using Image Lab (Bio-Rad Laboratories, Hercules, CA, USA) and normalized to total protein (Ponceau S staining).

### 2.9. Statistical Analysis 

A one-way analysis of variance (ANOVA) was used to test for differences in all assessed variables between dietary groups (EtOH-free HF: *n* = 8, 4 males and 4 females; LF + EtOH: *n* = 8, 5 males and 3 females; HF + EtOH: *n* = 7, 4 males and 3 females). The assumptions of normality and homogeneity of variances were verified prior to analysis. Equal variances were verified using descriptives and Levene’s test. Post-hoc analyses were performed with Bonferroni correction. Significance was set at α ≤ 0.05. PCR analyses were performed in PrimePCR Analysis Software Version 1 (Bio-Rad Laboratories, Hercules, CA, USA). All other analyses were performed in GraphPad Prism Version 8 (GraphPad Holdings, San Diego, CA, USA). All data are presented as means ± standard deviations.

## 3. Results

### 3.1. Alcohol Consumption, Body Weights, and Muscle Weights

Alcohol consumption (grams of EtOH consumed per kg body weight) across the treatment groups over the duration of the experiment were similar, irrespective of whether the data were analyzed per sex or combined (Supplemental Table S2). Final body weights after 6 weeks of feeding were not significantly different across treatment groups (Supplemental Table S3). Weight of the gastrocnemius muscle normalized to body weight or tibia length was not significantly different across treatment groups (Supplemental Table S4).

### 3.2. Myoglobin Concentrations

**Figure 1:**
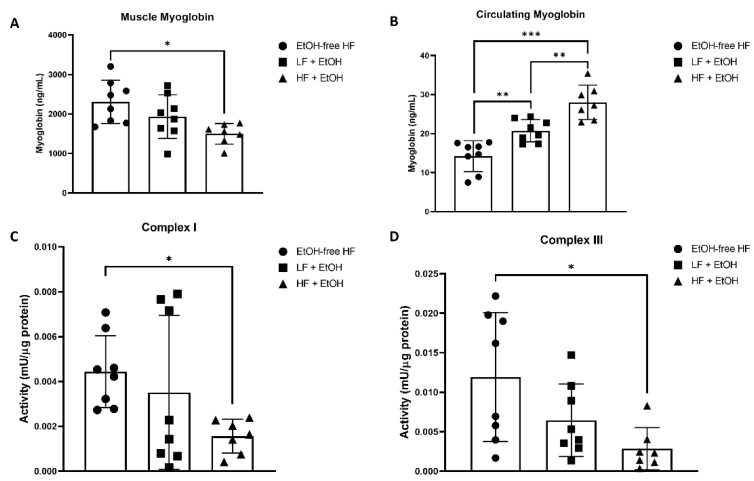
Myoglobin concentrations in (**A**) skeletal muscle and (**B**) plasma from mice fed an alcohol-free high-fat diet (EtOH-free HF), a low-fat diet including alcohol (LF + EtOH), or a high-fat diet including alcohol (HF + EtOH). Skeletal muscle mitochondrial electron transport chain activity in isolated mitochondria measured for (**C**) Complex I and (**D**) Complex III. * = *p* < 0.05; ** = *p* < 0.01; *** = *p* < 0.001.

Mice fed a HF + EtOH diet had significantly lower myoglobin concentrations in muscle compared to mice fed an EtOH-free HF diet (EtOH-free HF: 2307.59 ± 510.32 ng/mL, HF + EtOH: 1498.60 ± 242.22 ng/mL, *p* = 0.01) (Figure 1A). Circulating myoglobin levels were significantly higher in mice fed EtOH with either a LF (20.74 ± 2.65 ng/mL) or HF diet (28.00 ± 4.10 ng/mL), compared to mice fed an EtOH-free HF diet (14.23 ± 3.69 ng/mL) (*p* = 0.007 and *p* < 0.001, respectively) (Figure 1B). Circulating levels of myoglobin were also significantly higher in HF + EtOH diet fed mice compared to LF + EtOH diet fed mice (*p* = 0.004).

### 3.3. Electron Transport Chain Activity and Expression

The activity of Complex I of the electron transport chain was significantly reduced by 35% in muscles of mice fed a HF + EtOH diet relative to mice fed an EtOH-free HF diet (*p* = 0.045) (Figure 1C). Similarly, Complex III activity was significantly reduced by 24% in HF + EtOH diet fed mice relative to EtOH-free HF diet fed mice (*p* = 0.02) (Figure 1D).

**Figure 2:**
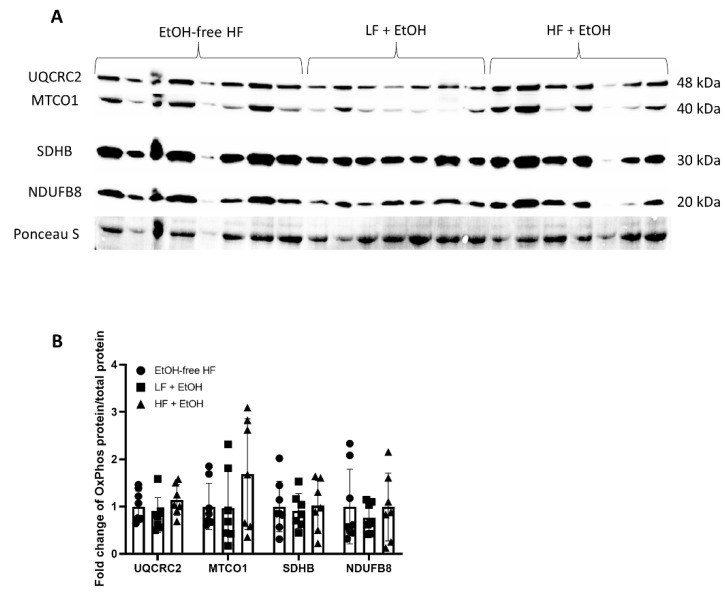
Protein expression of OXPHOS proteins in skeletal muscle homogenates from mice fed an alcohol-free high-fat diet (EtOH-free HF), a low-fat diet including alcohol (LF + EtOH), or a high-fat diet including alcohol (HF + EtOH). Protein expression determined by (**A**) Western blot, normalized to total protein (Ponceau S staining). (**B**) Fold change of protein expression.

Despite the decrease in Complex I and Complex III activity, the protein levels of the Complex I subunit, NADH:ubiquinone oxidoreductase subunit B8 (NDUFB8), as well as the Complex III subunit, ubiquinol-cytochrome C reductase core protein 2 (UQCRC2), were not significantly different in muscles of mice fed either a LF + EtOH or HF + EtOH diet, relative to the EtOH-free HF diet (Figure 2). Likewise, the protein levels of the Complex II subunit, succinate dehydrogenase complex iron sulfur subunit B (SDHB), and the Complex IV subunit, mitochondrially encoded cytochrome C oxidase I (MTCO1), also did not vary between groups.

### 3.4. Oxidative Stress and Antioxidant Markers

**Figure 3:**
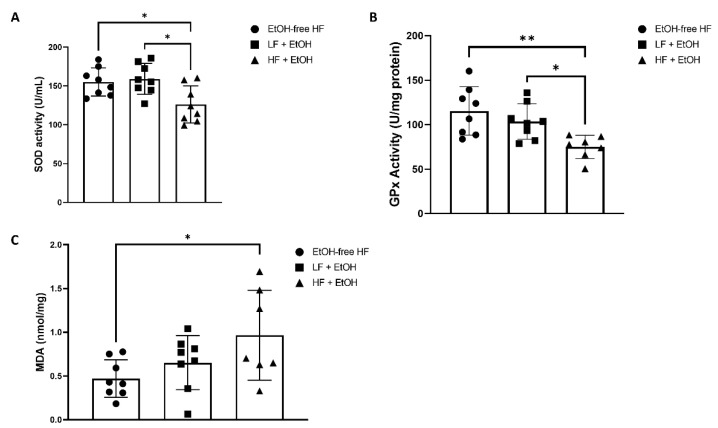
(**A**) Superoxide dismutase (SOD) activity and (**B**) glutathione peroxidase (GPx) activity measured as assays of antioxidant activity in skeletal muscle homogenates from mice fed an alcohol-free high-fat diet (EtOH-free HF), a low-fat diet including alcohol (LF + EtOH), or a high-fat diet including alcohol (HF + EtOH). (**C**) Malondialdehyde (MDA) levels measured as an index of lipid peroxidation. * = *p* < 0.05; ** = *p* < 0.01.

SOD activity was 19% lower in muscles of mice fed a HF + EtOH diet relative to the EtOH-free HF diet (*p* = 0.01) (Figure 3A). When compared to the LF + EtOH diet, SOD activity was 21% lower in HF + EtOH diet fed mice as well (*p* = 0.03). In addition, the antioxidant activity of glutathione-dependent peroxidases was significantly diminished by 35% in mice fed a HF + EtOH diet relative to the EtOH-free HF diet (*p* = 0.004). GPx activity was also 28% lower in mice fed a HF + EtOH diet compared to the LF + EtOH diet (*p* = 0.04) (Figure 3B). MDA, a marker of lipid peroxidation, was significantly increased two-fold in muscles of mice fed a HF + EtOH diet (*p* = 0.04), compared to mice fed an EtOH-free HF diet (Figure 3C).

**Figure 4:**
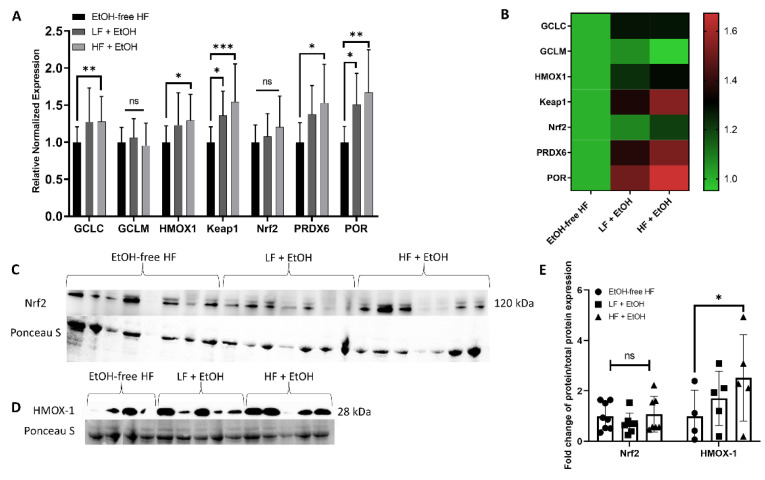
(**A**) Relative fold change of mRNA expression detected by real-time PCR. Gene expression measured in skeletal muscle homogenates of male (M) and female (F) mice fed an alcohol-free high-fat diet (EtOH-free HF) (*n* = 8), a low-fat diet including alcohol (LF + EtOH) (*n* = 8), or a high-fat diet including alcohol (HF + EtOH) (*n* = 7). (**B**) Heatmap analysis of expression of antioxidant genes, color legend represents calculated relative expression of different dietary groups compared to EtOH-free HF group. Expression of select corresponding proteins measured by Western blot of (**C**) Nrf2 and (**D**) Hmox-1, normalized to total protein assessed by Ponceau S staining. (**E**) shows fold change of protein expression. ns = not significant; * = *p* < 0.05; ** = *p* < 0.01; *** = *p* < 0.001.

The expression profiles of several genes encoding antioxidants, including GCLC, GCLM, HMOX1, Keap1, Nrf2, PRDX6, and POR, were assayed in gastrocnemius muscles. Interestingly, mice fed a HF + EtOH diet demonstrated significant elevations in GCLC (1.27-fold, *p* = 0.008), HMOX1 (1.23-fold *p* = 0.02), Keap1 (1.55-fold, *p* = 0.006), PRDX6 (1.53-fold, *p* = 0.02), and POR (1.67-fold, *p* = 0.001) relative to the EtOH-free HF diet. The expression of Keap1 (1.36-fold, *p* = 0.04) and POR (1.51-fold, *p* = 0.03) were also significantly elevated in mice fed a LF + EtOH diet relative to the EtOH-free HF diet (Figure 4A,B).

Consistent with the gene expression changes, there was no significant difference in Nrf2 protein levels between groups. However, mice fed a HF + EtOH diet had significantly increased HMOX-1 protein levels relative to EtOH-free HF diet fed mice (2.52-fold, *p* = 0.04) (Figure 4C–E).

### 3.5. Alcohol-Associated Regulators of Mitochondrial Function

**Figure 5:**
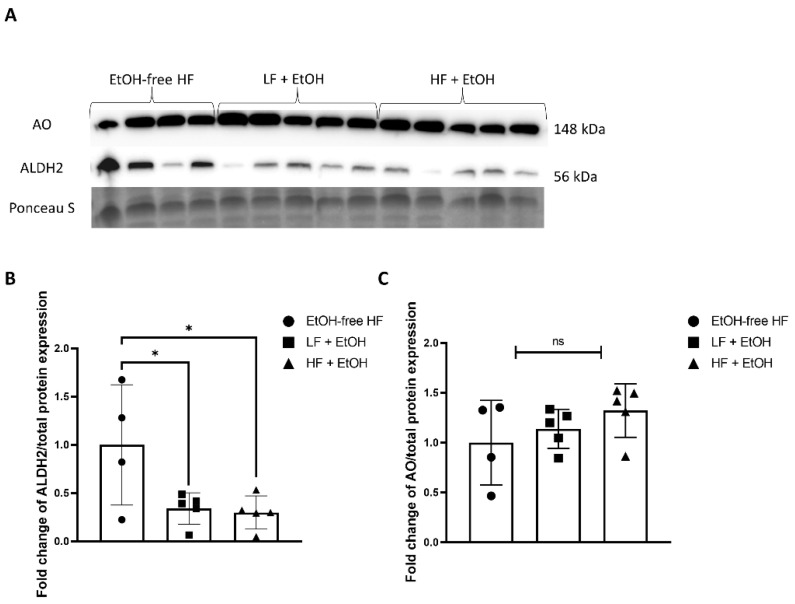
Protein expression of ALDH2 and AO in skeletal muscle homogenates from mice fed an alcohol-free high-fat diet (EtOH-free HF), a low-fat diet including alcohol (LF + EtOH), or a high-fat diet including alcohol (HF + EtOH) determined by (**A**) Western blot, normalized to total protein (Ponceau S staining). Fold change of (**B**) ALDH2 expression and (**C**) AO expression, ns = not significant; * = *p* < 0.05.

Gastrocnemius muscle aldehyde dehydrogenase 2 (ALDH2) protein expression was significantly lower in mice fed both a LF + EtOH diet (0.34-fold, *p* = 0.045) and a HF + EtOH diet (0.30-fold, *p* = 0.03) relative to EtOH-free HF diet (Figure 5A,B). There was no significant difference in aldehyde oxidase (AO) protein expression between groups (Figure 5A,C).

## 4. Discussion

In the present work, we sought to examine how different dietary compositions influence alcohol’s effects on skeletal muscle mitochondrial electron transport chain activity and oxidative stress. We found that a combination of HF-feeding and EtOH led to significantly reduced myoglobin concentrations in skeletal muscle and significantly increased myoglobin concentrations in plasma, which may suggest myoglobin leakage into the bloodstream. Increased myoglobin levels in circulation is a known feature of mitochondrial myopathy [36]. In fact, repeated alcohol intake has been shown to cause acute muscle damage and increase serum myoglobin levels [36].

Furthermore, mice fed a HF + EtOH diet had significantly lower skeletal muscle Complex I and Complex III activity relative to an EtOH-free HF diet. The expression of several antioxidant enzymes was also significantly increased in HF + EtOH diet fed mice relative to the EtOH-free HF diet. The up-regulation of antioxidant genes was accompanied by reductions in SOD and GPx activity and increases in the lipid peroxidation marker, MDA. SOD constitutes the first line of defense against ROS, converting superoxide into oxygen and hydrogen peroxide, and GPx converts hydrogen peroxide into water [37]. Lower SOD and GPx activity are thought to be a response to chronic systemic oxidative stress [38,39]. Thus, the combination of HF diet and EtOH consumption may result in a compensatory rise in antioxidant expression in response to increased ROS production. However, the antioxidant systems are likely overwhelmed, resulting in reduced antioxidant activity and increased oxidative stress.

The increases in several of the antioxidant genes that accompanied the HF + EtOH diet can potentially be explained by increased oxidative stress. For example, GCLC, the rate limiting enzyme for glutathione synthesis, is transcriptionally controlled by oxidative stress [40]. Likewise, HMOX1 is a stress-induced isoform of heme oxygenases, and its expression is increased after oxidative stress [41]. Further, PRDX6 has GPx activity, and an elevation in both GCLC and PRDX6 may be an attempt to restore the diminished GPx activity which was found [42]. Interestingly, the expression of the assessed antioxidant enzymes is regulated by Nrf2, a transcription factor that coordinates stress-inducible activation of antioxidant systems [43]. While Nrf2 gene expression and protein levels were unaffected in HF + EtOH diet fed mice, it is important to note that Nrf2 activation is controlled by various factors, including Keap1-mediated degradation and post-transcriptional regulation by microRNAs, binding partners, alternative splicing, epigenetic modifications, phosphorylation, and subcellular localization [43]. A complete understanding of the role of Nrf2 in alcohol and HF diet-induced mitochondrial dysfunction and oxidative stress will require future integrated considerations of the complex Nrf2 regulatory network.

The reduced Complex activity in HF + EtOH diet-fed mice was not accompanied by any reductions in OXPHOS proteins compared to the EtOH-free HF diet. The seemingly paradoxical observation of unaltered or elevated mitochondrial content despite reduced respiration is similar to previous work in HF-diet fed mice [14], which is thought to be the consequence of increased mitochondrial biogenesis to account for dysfunctional or damaged mitochondria [44]. There is also evidence that chronic alcohol consumption can reduce mitochondrial fusion via reduced mitofusin (Mfn)1 expression [45]. Moreover, ethanol-induced mitochondrial fragmentation, mediated by the mitochondrial fission factor dynamin-related protein 1 (DRP-1), has been documented [46]. In addition to mitochondrial dynamics, toxic agents including alcohol and HF can also induce oxidative stress which promotes post-translational modification and subsequent inactivation of mitochondrial proteins [47]. Interestingly, one of the proteins that has been shown to be post-translationally modified and inactivated by hepatotoxic-associated ROS is GPx [48], and in the present study, we observed reduced GPx activity in HF + EtOH diet fed mice.

Another mechanism thought to be involved in alcohol-induced mitochondrial dysfunction is related to alcohol metabolism. Alcohol is oxidized to aldehydes and ketones by alcohol dehydrogenase (ADH) and the mitochondrial enzyme, aldehyde dehydrogenase 2 (ALDH2) [49]. Heavy alcohol consumption has been shown to downregulate ALDH2 expression [50]. In the present study, ALDH2 expression was also reduced in alcohol-fed mice. Importantly, ALDH2 also plays an important role in metabolizing other toxic aldehydes, including the lipid peroxidation product 4-hydroxy-2-nonenal (4-HNE) [51,52]. Alcohol exposure to ALDH2-knockout mice results in greater mitochondrial dysfunction [53], and ALDH2 overexpression has been shown to attenuate alcohol-induced cardiac dysfunction [54]. In addition, ALDH2 has also been shown to ameliorate HF diet-induced cardiomyopathy as well [55], and ALDH2-mutant mice with lower ALDH2 activity fed a HF diet exhibit greater metabolic syndrome and cardiac dysfunction [56,57]. Thus, the reduction in ALDH2 may be a factor contributing to the increased oxidative stress and diminished electron transport chain complex activity in mice exposed to HF and alcohol.

Notably, in this study and in previous work [58], no interactions or main effects for alcohol consumption between diets were found; thus, equivalent amounts of alcohol were consumed between diets. Differences between dietary groups in this study are therefore likely not due to differences in alcohol consumption, and instead due to the differing macronutrient compositions of the diets. Interestingly, when comparing mice fed LF + EtOH diets and HF + EtOH diets, there were some notable differences. Circulating myoglobin levels were significantly higher in HF + EtOH diet fed mice than LF + EtOH diet fed mice, and both SOD and GPx activity were diminished in muscles of HF + EtOH diet fed mice compared to LF + EtOH diet fed mice. Thus, a HF diet may exacerbate the effects of alcohol on skeletal muscle. However, a limitation of our current work is the lack of an EtOH-free LF diet for comparison.

While not assessed presently, the negative effects of the HF diet may be due to an increase in hepatic fat accumulation and thereby alcoholic liver disease, which can further contribute to the negative actions of alcohol on skeletal muscle [59,60]. Alternatively, the source of the excess fat may have also contributed to the presently observed effects, as corn oil exacerbates alcohol-related liver injury as well [61]. Notably, the Lieber-DeCarli diet is high in monounsaturated fat and low in saturated fatty acids [32,62]. Unsaturated fats have been shown to enhance alcohol-induced liver injury, and dietary saturated fat has been shown to play a protective role against alcohol-induced liver injury [63,64,65,66,67]. Skeletal muscle also appears sensitive to the macronutrient profile of the diet during chronic alcohol consumption, although further work is required to determine the direct role of hepatic disease on these outcomes. Future research should also investigate the effect of alcohol and saturated fat, in comparison to unsaturated fat, on skeletal muscle mitochondrial function.

In conclusion, consumption of a HF diet may exacerbate the negative effects of alcohol on mitochondrial function. The concomitant intake of a HF diet and alcohol may induce a metabolic myopathy, overwhelm antioxidant systems, and result in increased levels of oxidative stress. The HF and alcohol-mediated effects may be driven by alcohol-induced downregulation of ALDH2 expression.

## Figures and Tables

**Table 1:** Dietary macronutrient breakdown.

Diet Name	Carbohydrates	Fat	Protein	Alcohol
EtOH-free HF Diet	47	35	18	0
HF + EtOH Diet	15	35	18	32
LF + EtOH Diet	38	12	18	32

Table 1 shows the macronutrient distribution of each of the diets used herein. EtOH-free HF: Alcohol-free high-fat; HF + EtOH: high-fat with alcohol; LF + EtOH: low-fat with alcohol. Numbers are % of total kcals of the diet. The concentration of alcohol was gradually increased in a stepwise manner until reaching 32%. Maltose dextrin was used in the EtOH-free HF diet for isocaloric matching of all diets.

**Table 2:** Primers for quantification of antioxidant mRNAs by qPCR.

mRNA	5′—————3′	Forward/Reverse	Amplicon Size (Base Pairs)
GCLC	AACACAGACCCAACCCAGAG TGGCACATTGATGACAACCT	Forward Reverse	143
GCLM	TGTGTGATGCCACCAGATTT GCTTCAATGTCAGGGATGCT	Forward Reverse	137
HMOX1	TTACCTTCCCGAACATCGAC TCCTCTGTCAGCATCACCTG	Forward Reverse	172
Keap1	GGCAGGACCAGTTGAACAGT ATCACTGTCCGGGTCATAGC	Forward Reverse	188
Nrf2	AGCAAGTTTGGCAGGAGCTA TTCTTTTTCCAGCGAGGAGA	Forward Reverse	164
PRDX6	AAACACCCACGGAAAAGTTG GTTTCTTGTCAGGGCCAAAA	Forward Reverse	153
POR	GTCTTCTGCATGGCCACATA TGGCGTTGAAGTGCTCATAG	Forward Reverse	151
B2M ^1^	TGGTCTTTCTGGTGCTTGTC GGGTGGAACTGTGTTACGTAG	Forward Reverse	112

^1^ Reference mRNA. GCLC: Glutamate-Cysteine Ligase Catalytic Subunit; GCLM: Glutamate-Cysteine Ligase Modifier Subunit; HMOX1: Heme Oxygenase 1; Keap1: Kelch-like ECH-associated protein 1; Nrf2: Nuclear factor-erythroid factor 2-related factor 2; PRDX6: Peroxiredoxin 6; POR: Cytochrome P450 Oxidoreductase; B2M: Beta-2 microglobulin.

## Data Availability

The datasets generated during and/or analyzed during the current study are available from the corresponding author on reasonable request.

## Supplementary Materials

The following supporting information can be downloaded at: https://www.mdpi.com/article/10.3390/nu14051016/s1, Table S1: Dietary Ingredient Breakdown; Table S2: Average Alcohol Intake; Table S3: Body Weights; Table S4: Muscle Weights.
