# Supplementary material for: High-Fat Diet Augments the Effect of Alcohol on Skeletal Muscle Mitochondrial Dysfunction in Mice
Source: Nutrients. 2022 Feb 28;14(5):1016. doi: 10.3390/nu14051016 (PMC8912391; doi:10.3390/nu14051016)
Supplement: Supplementary file 1 [file nutrients-14-01016-s001.zip › nutrients-1577731-supplementary.pdf]

Supplementary Material

Supplemental Table S1. Dietary Ingredient Breakdown

Supplemental Table S2. Average Alcohol Intake

Supplemental Table S3. Body Weights

Supplemental Table S4. Muscle Weights

Supplemental Tables

Supplemental Table S1. Dietary Ingredient Breakdown

Diet Name	EtOH-free HF	LF + EtOH	HF + EtOH
**Ingredient:**	**g/Liter**	**g/Liter**	**g/Liter**
**Casein (80 Mesh)**	41.40	41.40	41.40
**DL-Methionine**	0.30	0.30	0.30
**L-Cystine**	0.50	0.50	0.50
**Cellulose**	10.00	10.00	10.00
**Maltose Dextrin**	115.20	93.56	35.56
**Corn Oil**	8.50	2.50	8.50
**Olive Oil**	28.40	8.40	28.40
**Safflower Oil**	2.70	2.70	2.70
**Mineral Mix**[1]	8.75	8.75	8.75
**Vitamin Mix**[2]	2.50	2.50	2.50
**Choline Bitartrate**	0.53	0.53	0.53
**Xanthan Gum**	3.00	3.00	3.00
**Alcohol (32% kcal)**	0	48.10	48.10

[1]Mineral Mix: Ca (5,000 g/kg), P (1,561 g/kg), K (3,600 g/kg), Na (1,019 g/kg), Cl (1,571 g/kg), S (300 g/kg), Mg (507 g/kg), Fe (35 g/kg), Cu (6 g/kg), Mn (10 g/kg), Zn (30 g/kg), Cr (1 g/kg), I (0.2 g/kg), Se (0.15 g/kg), F (1 g/kg), B (0.5 g/kg), Mo (0.15 g/kg), Si (5 g/kg), Ni (0.5 g/kg), Li (0.1 g/kg), V (0.1 g/kg). [2]Vitamin Mix: Niacin (3 g/kg), Calcium Pantothenate (1.6 g/kg), Pyridoxine HCl (0.7 g/kg), Thiamine HCl (0.6 g/kg), Riboflavin (0.6 g/kg), Folic Acid (0.2 g/kg), Biotin (0.02 g/kg), Vitamin E Acetate (500 IU/g, 15 g/kg), Vitamin B12 (0.1%, 2.5 g/kg), Vitamin A Palmitate (500,000 IU/g, 0.8 g/kg), Vitamin D3 (400,000 IU/g, 0.25 g/kg), Vitamin K1/Dextrose Mix (10 mg/g, 7.50 g/kg), Sucrose (967.23 g/kg).

Supplemental Table S2. Average Alcohol Intake

	EtOH-free HF	LF + EtOH	HF + EtOH
**Overall average**	0	18.6 ± 4.6	17.6 ± 4.3

Supplemental Table S3. Body Weight

Body Weight (g)

	EtOH-free HF	LF + EtOH	HF + EtOH
**Overall average**	26.24 ± 4.96	27.48 ± 2.79	27.41 ± 2.83

Supplemental Table S4. Muscle Weight

Gastrocnemius Weight (mg tissue/gram BW)

	**EtOH-free HF**	**LF + EtOH**	**HF + EtOH**
**Overall average**	4.64 ± 0.55	4.45 ± 0.29	4.54 ± 0.49
